# How does nitrogen shape plant architecture?

**DOI:** 10.1093/jxb/eraa187

**Published:** 2020-04-11

**Authors:** Le Luo, Yali Zhang, Guohua Xu

**Affiliations:** 1 State Key Laboratory of Crop Genetics and Germplasm Enhancement, Nanjing Agricultural University, Nanjing, China; 2 China MOA Key Laboratory of Plant Nutrition and Fertilization in Lower-Middle Reaches of the Yangtze River, Nanjing, China; 3 Michigan State University, USA

**Keywords:** Amino acids, ammonium, architecture, flowering time, nitrate, panicle structure, phytohormones, shoot branching, tillering, transcription factor, transporter

## Abstract

Plant nitrogen (N), acquired mainly in the form of nitrate and ammonium from soil, dominates growth and development, and high-yield crop production relies heavily on N fertilization. The mechanisms of root adaptation to altered supply of N forms and concentrations have been well characterized and reviewed, while reports concerning the effects of N on the architecture of vegetative and reproductive organs are limited and are widely dispersed in the literature. In this review, we summarize the nitrate and amino acid regulation of shoot branching, flowering, and panicle development, as well as the N regulation of cell division and expansion in shaping plant architecture, mainly in cereal crops. The basic regulatory steps involving the control of plant architecture by the N supply are auxin-, cytokinin-, and strigolactone-controlled cell division in shoot apical meristem and gibberellin-controlled inverse regulation of shoot height and tillering. In addition, transport of amino acids has been shown to be involved in the control of shoot branching. The N supply may alter the timing and duration of the transition from the vegetative to the reproductive growth phase, which in turn may affect cereal crop architecture, particularly the structure of panicles for grain yield. Thus, proper manipulation of N-regulated architecture can increase crop yield and N use efficiency.

## Teaser

Nitrogen regulation of the relationship between plant height and tillering and panicle structure occurs at different developmental stages through integration of the synthesis and distribution of multiple phytohormones.

## Introduction

Nitrogen (N) is quantitatively the most important mineral nutrient in plants. N is acquired as nitrate (NO_3_^−^) and/or ammonium (NH_4_^+^) from soil (Xu *et al.*, 2012). High-yield cultivation relies heavily on the use of N fertilizers. Excessive application of N fertilizers not only causes energy waste and increases production costs but also aggravates soil acidification and water eutrophication, as well as contributing to greenhouse gas emissions (Guo *et al.*, 2010; Sutton *et al.*, 2011). Therefore, there is an urgent need to breed crop varieties that use N efficiently in order to minimize N input for the sake of sustainable plant production.

For efficient acquisition of N from soil, plants have developed sophisticated regulatory mechanisms of root development and N transport. Several recent reviews (Forde *et al.*, 2014; Giehl *et al.*, 2014; Kiba *et al.*, 2016; O’Brien *et al.*, 2016; Xuan *et al.*, 2017; Taleski *et al.*, 2018; Yang *et al.*, 2019) and two reviews in this Special Issue (Liu *et al.*, 2020; Jia and von Wirén, 2020) have described root adaptation to altered N supply and the prospects for genetically engineering ideal root phenotypes. Meanwhile, the growth and development of aboveground plant parts are systematically regulated by N status (Chen *et al.*, 2016; O’Brien *et al.*, 2016; Xuan *et al.*, 2017). Architectural features that are affected by N, such as plant height, branches, and panicles not only affect yield but also determine N distribution in various organs as well as the efficiency of N use (Hu *et al.*, 2015; Chen *et al.*, 2017 ). Although the basic regulatory mechanisms of plant architecture have been characterized (Wang and Li, 2008; Xing and Zhang, 2010; Wang *et al.*, 2018a), reports concerning the N regulation of plant architecture, particularly by different forms of N at the cellular and molecular levels, are limited and dispersed in the literature. In this review, we summarize how the N supply shapes plant architecture and discuss the possible relationships between plant architecture, growth duration, and N use efficiency (NUE), mainly in cereal crops.

## Nitrogen regulation of growth and development in different phases

The hormonal and genetic control of plant architecture, including shoot apical meristem (SAM) activity, axillary meristem formation and elongation, inflorescence structure, and plant height, has been characterized in Arabidopsis, rice, pea, maize, and tomato (Wang and Li, 2008; Xing and Zhang, 2010; Wang *et al.*, 2018a). During developing phases, the plant architecture changes in several aspects, such as stem elongation, branch development, stem and leaf angle, and inflorescence development (Wang *et al.*, 2018a). For cereal crops, the branch number and panicle structure (inflorescence) are two of the most important traits that directly determine the grain yield (Kyozuka *et al.*, 2014). The number of branches (or tillers in rice and wheat) is determined by the initiation of axillary meristem and thereafter via the elongation of axillary buds (Bennett and Leyser, 2006). The mechanism controlling axillary bud outgrowth in apical dominance has been extensively studied; auxin is the main player involved in axillary bud regulation (Teale *et al.*, 2006). The panicle structure is also determined by meristem activity. Floral meristem is the final phase wherein the meristem activity ceases. In grass species, the basic panicle structure is determined by spikelets, the small branches for producing flowers (Itoh *et al.*, 2005; Kellogg *et al.*, 2013). It has been shown that the inflorescence in rice is mainly determined by the floral meristem, which controls the timing of phase transition from the vegetative to the reproductive stage, thereby influencing panicle size (Itoh *et al.*, 2005). Early transition decreases the number and length of branches and panicles, while delayed transition results in more and longer branches as well as larger panicles (Kyozuka *et al.*, 2014).

The plant architecture is greatly influenced by aspects of the growth environment, particularly the duration and intensity of light, and supplies of nutrients and water (Kudoyarova *et al.*, 2015; de Wit *et al.*, 2016; Feng *et al.*, 2016; Wang *et al.*, 2018a). N is one of the major determinants of plant growth and development that affect the major components of plant architecture such as tiller number and panicle structure (Ladha *et al.*, 1998; Zhang et al., 2009, 2017; Tian *et al.*, 2017; Wang *et al.*, 2018a; Yi *et al.*, 2019; Yang *et al.*, 2019). The plant N uptake rate varies during different growth and development stages. In rice, total N accumulation rapidly increases during the vegetative and early reproductive stages, then reaches a plateau before declining slightly during grain filling and ripening (Hashim *et al.*, 2015). The root uptake rate and concentration of N during early growth stages are critical for forming effective tillers. During the ripening stage, the N that is used for grain formation and seed filling is transferred mainly from culms and leaves (Hashim *et al.*, 2015). Therefore, varying the rate of N application at different stages (sowing, tillering, panicle initiation, and heading) can alter the yield components of rice. The N demand for forming effective tillers and for grain filling (weight) may vary among different varieties, probably due to their differences in growth and development (Thu *et al.*, 2014).

**Fig. 1.**
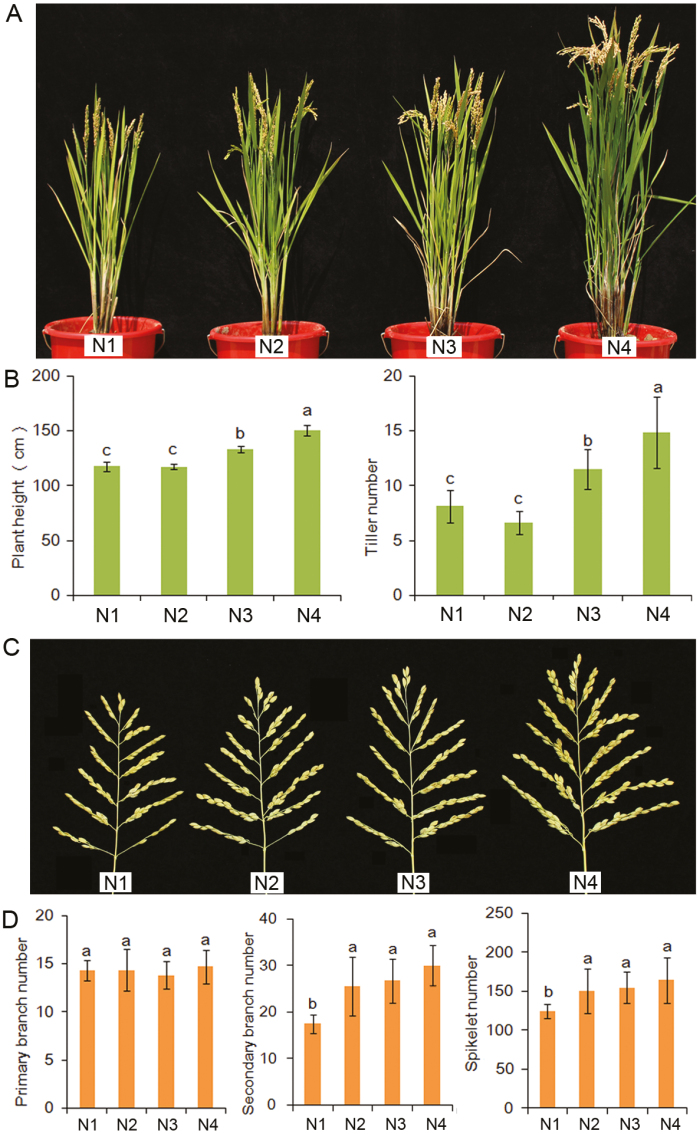
Different responses of the major architecture components to nitrogen (N) fertilization in rice. A *japonica* rice cultivar (cv. 92-10geng) was grown in a paddy field with four different levels of N fertilizer applied and was transferred at the late grain-filling stage into pots for photographing and measurement of the plant structure. (A) The phenotypes of plants supplied with four different N levels. N1, lowest application (75 kg N ha^–1^); N2, low application (150 kg N ha^–1^); N3, moderate application (250 kg N ha^–1^); N4, high application (350 kg N ha^–1^). (B) Plant height and effective tiller number. (C) The shape of entire panicles. (D) Numbers of primary branches, secondary branches, and spikelets. Data in (B) and (D) are mean ±SD (*n*≥8).Means with different letters are significantly different (*P*<0.05).

Changes in plant architecture in response to N supply may vary among plant species and even accessions of the same species. We have observed natural variation in the response of the different components of plant architecture to N fertilization among rice accessions in a core collection grown in a paddy field (data not shown). Nevertheless, N limitation suppresses rice growth, decreases height, and limits tiller number (Fig. 1A, B). Notably, the N demand for maintaining height and tiller number is not the same as that for the branch number of spikelets, and the growth of secondary branches rather than primary branches is sensitive to the supply of N (Fig. 1C, D).

In general, the effect of the level of N supply on plant height can be predicted, whereas its effect on other architecture components, such as tiller number, filled grains per panicle, 1000-grain weight, and grain yield, is complicated. Sufficient N supply stimulates shoot elongation and ensures that the plant will reach the expected height in both rice and wheat (Wu *et al.*, 2020), while excess N prevents secondary cell wall formation, resulting in poor lodging resistance (Wu *et al.*, 2017; Zhang *et al.*, 2017). The tiller number is affected by the N supply level and growth stage. The effective tiller number can be increased by increasing the N supply to an appropriate level and can be decreased by the excessive application of N (Haque *et al.*, 2016). In rice, N deficiency suppresses bud elongation rather than initiation (Luo *et al.*, 2017). The most critical time for N fertilization for rice grain yield is at the panicle initiation stage (Yoshida *et al.* 2006). The N supply affects inflorescence development, panicle length, and the number of flowers per panicle (Yoshida *et al.* 2006; Makino, 2011). In wheat, N accumulation at anthesis was found to be positively correlated with the onset of flag-leaf senescence, and thus total N accumulated at anthesis is an important trait for enhancing grain yield and NUE under low to moderate N supply (Nehe *et al.*, 2018).

For efficient use of light, water, and nutrient resources, it is imperative that plants have the phenotypic plasticity to be able to adapt to varied environmental conditions. Interestingly, super-high-yield rice cultivars show high morphological acclimation in leaf dispersion and orientation to different agronomic practices, including N application (Wang *et al.*, 2019a). The efficient phenotypic adaptation of rice is coordinated with improved N uptake and assimilation; the shoot photosynthetic productivity of a given rice phenotype is closely and positively related to leaf N concentration and total N accumulation (Wang *et al.*, 2019a).

## Nitrate in the regulation of shoot branching and flowering

The mechanism of N regulation of plant architecture has been partially elucidated during the past decades. In wheat, He *et al.* (2015) isolated a NO_3_^−^-inducible and cereal-specific NAC (NAM, ATAF, and CUC) transcription factor, TaNAC2-5A. Limited NO_3_^−^supply enhances the expression of *TaNAC2-5A* in shoots and roots. TaNAC2-5A can directly bind to the promoter regions of the genes encoding NO_3_^−^ transporters and glutamine synthetase, consequently enhancing N acquisition and assimilation. Overexpression of TaNAC2-5A can increase tiller numbers, spikelet number, and 1000-grain weight, resulting in higher grain yield (He *et al.*, 2015). In rice, OsMADS57, a MADS-box transcription factor whose expression is enhanced by NO_3_^−^ supply, interacts with TEOSINTE BRANCHED1 (TB1) and targets Dwarf14 (D14) to control the outgrowth of axillary buds (Guo *et al.*, 2013; Huang *et al.*, 2019a). OsMADS57 can also bind to the CArG motif (CATTTTATAG) within the promoter of *OsNRT2.3a* that functions in NO_3_^−^ translocation; knockout of OsMADS57 suppresses the distribution of NO_3_^−^ from root to shoot (Tang *et al.*, 2012; Huang *et al.*, 2019a). These results suggest that OsMADS57 may participate in NO_3_^−^-regulated tiller bud outgrowth of rice plants.

Some NO_3_^−^ transporters have been reported to participate in the modulation of plant architecture (tiller number and panicle architecture), mainly through changing N uptake and translocation to reproductive organs. Overexpression of *OsNRT2.3b*, but not of *OsNRT2.3a*, increases panicle size parameters including panicle length, number of primary and secondary rachises, number of seeds per panicle, and seed-setting rates under different N treatments (Fan *et al.*, 2016). Chen et al. (2016, 2017) reported that overexpression of a high-affinity NO_3_^−^ transporter gene, *OsNRT2.1*, driven either by the promoter of *OsNAR2.1* (encoding a nitrate transport accessory protein) or by its own promoter can increase post-anthesis N uptake and translocation from vegetative organs to grains, resulting in greater panicle length and seed set, more grains per panicle, and higher grain yield.

Several members of the NO_3_^−^ and peptide transporter family (NPF) in rice have been characterized with regard to their functions in regulating shoot branching and panicle structure. OsNPF7.7 has two splicing variants, OsNPF7.7-1 and OsNPF7.7-2, that show similar expression responses to N in axillary buds (Huang *et al.*, 2018). Enhanced expression of *OsNPF7.7-1* and *OsNPF7.7-2* increases NO_3_^−^ and NH_4_^+^ influx, respectively, while both OsNPF7.7-1 and OsNPF7.7-2 promote the outgrowth of axillary buds and increase the numbers of tillers, effective panicles, and filled grains per plant, resulting in higher grain yield (Huang *et al.*, 2018). In addition, both OsNPF7.1 and OsNPF7.4 function in NO_3_^−^ uptake, but they show opposite expression patterns in axillary buds (Huang *et al.*, 2019b). Overexpression of *OsNPF7.1* or knockout of *OsNPF7.4* can increase axillary bud outgrowth, especially for the second bud, and subsequently tiller number in rice. Moreover, OsNPF7.2, a low-affinity nitrate transporter, can positively alter cell division in tiller buds to increase tiller number and grain yield (Wang *et al.*, 2018b).

Very recently, the *indica* allele of the nitrate reductase gene *OsNR2*, which encodes a NADH/NADPH-dependent nitrate reductase, has been shown to promote NO_3_^−^ uptake via feed-forward interaction with a NO_3_^−^ transporter, OsNRT1.1B, thereby enhancing rice yield potential and NUE (Gao *et al.*, 2019). Notably, effective tiller number is increased in Nipponbare plants expressing *indica OsNR2* (cv. 9311) and decreased by reduced *OsNR2* expression, probably via alteration of the expression of rice *OsTB1*, a gene controlling tiller bud formation and elongation (Gao *et al.*, 2019). The feed-forward interaction of OsNR2 and OsNRT1.1B may explain the effect of OsNRT1.1B in altering tiller number in rice (Hu *et al.*, 2015).

Another aspect of nitrate-dependent regulation of plant architecture may be associated with flowering time. N fertilization influences the length of different growth phases and results in varied architecture, even within the same genotype (Leng *et al.*, 2020; Hall *et al.*, 2014). The transition from the vegetative to the reproductive phase is the end of leaf generation on the main stem; this influences the axillary meristem number. The rice branch number is commonly increased by late flowering and decreased by early flowering (Leng *et al.*, 2020). Excess N application commonly causes a delay in the flowering time, resulting in later ripening. In Arabidopsis, the influence of N supply on flowering time has been well characterized (Castro Marín *et al.*, 2011; Vidal *et al.*, 2014). Both extreme deficiency and excess of N result in postponement of flowering time (Lin and Tsay, 2017). SUPPRESSOR OF OVEREXPRESSION OF CONSTANS 1 (SOC1) serves as a central integrator for multiple flowering pathways in the SAM (Srikanth and Schmid, 2011). It has been shown that NO_3_^–^ acts at the SAM to regulate flowering time, and that SOC1 is required for the regulation of N-dependent flowering (Olas *et al.*, 2019). High nitrate can activate two AP2-type transcription factors, SCHLAFMUTZE (SMZ) and SCHNARCHZAPFEN (SNZ), via the gibberellin (GA) pathway to repress flowering time (Gras *et al.*, 2018). High N levels inhibit the activity of ferredoxin–NADP oxidoreductase (FNR1), leading to the induction of the blue-light receptor cryptochrome 1 (CRY1) and FNR1 nuclear degradation, which act in the N signal input pathway to affect central circadian clock gene expression and flowering time (Yuan *et al.*, 2016). It is an intriguing question whether there are common regulatory pathways of N-dependent alteration of flowering time and plant architecture in addition to their indirect effect on the plant architecture.

## Amino acids in the regulation of plant architecture

Plant roots can directly acquire a large portion of NH_4_^+^ and amino acids in addition to NO_3_^−^ in the soil, particularly under highly N-fertilized or irrigated paddy conditions. After absorption, most of the NH_4_^+^ and part of the NO_3_^−^ are assimilated to amino acids in the roots; therefore, the transportation and distribution of N inside plants occur mainly in the form of amino acids (Xu *et al.*, 2012). Although NH_4_^+^ could regulate root architecture (Liu and von Wirén, 2017), it is not clear whether NH_4_^+^ itself can directly regulate shoot architecture; however, several studies have shown that some transporters and synthetases of amino acids are directly involved in the regulation of plant architecture.

Amino acids are essential components of plant metabolism, not only as constituents of proteins but also as precursors of important secondary metabolites and as carriers of organic N between the organs of the plant (Dinkeloo *et al.*, 2018). Amino acids in the roots are transported from the cortex or endodermis cells to the vasculature to circumvent the Casparian strip and then translocated to the aboveground tissues (Dinkeloo *et al.*, 2018; Tegeder and Masclaus-Daubresse, 2018). Amino acid transporters play roles in amino acid uptake by roots, xylem and phloem loading, xylem–phloem transfer, and intracellular transport (Dinkeloo *et al.*, 2018). It has been recently shown that amino acid transporters function in the regulation of tiller growth and the entire plant architecture in addition to altering N distribution and NUE. In rice, blocking amino acid permease 3 (AAP3) can stimulate bud outgrowth and effective tiller number, leading to higher grain yield; in contrast, overexpressing OsAAP3 results in an enriched amount of amino acids and inhibition of bud outgrowth (Lu *et al.*, 2018). Amino acid permease 5 (OsAAP5) can also affect tiller number and grain yield through the regulation of cytokinin (CK) biosynthesis (Wang *et al.*, 2019b).

The synthesis of amino acids may be involved in regulating the plant architecture. We have shown that mutation of asparagine synthetase 1 (ASN1) in rice decreased the concentration of asparagine, while total N was unchanged (Luo *et al.*, 2019). Knockout of *OsASN1* suppressed tiller bud outgrowth and tiller number, suggesting that OsASN1 is involved in the regulation of rice development (Luo *et al.*, 2019). Rice cytosolic glutamine synthetase OsGS1;2, which is involved in the primary assimilation of NH_4_^+^ in roots, is also involved in the regulation of plant development (Funayama *et al.*, 2013; Ohashi et al., 2015, 2017). It has been demonstrated that OsGS1;2 contributes to tiller bud outgrowth by regulating N-dependent CK biosynthesis (Ohashi *et al.*, 2017).

In addition to their functions as basic compounds for growth and development, amino acids may function as signaling molecules (Dinkeloo *et al.*, 2018). For example, serine acts as a signal in brain tissue and in mammalian cancer cells. The biosynthesis of serine is highly active and restricted to proliferating cells of the primary meristem (Häusler *et al.*, 2014). Serine in the meristems has been suggested to regulate targets of rapamycin signaling in plants (Benstein *et al.*, 2013; Cascales-Minana et al., 2013; Menand *et al.*, 2002). Moreover, it is very likely that there are amino acid transceptors that are involved in the regulation of plant development (Dinkeloo *et al.*, 2018).

## Nitrogen-regulated small signaling peptides in plant development

Small signaling peptides have been identified in plant cell-to-cell communication and plant growth regulation (Czyzewicz *et al.*, 2013; Tavormina *et al.*, 2015; Oh *et al.*, 2018). Two small peptide families, CLAVATA3/EMBYO SURROUNDING REGION (CLE) and C-TERMINALLY ENCODED PEPTIDE (CEP), function in both local N-status-dependent signaling and systemic N signaling (Okamoto *et al.*, 2016; de Bang *et al.*, 2017; Taleski *et al.*, 2018). CEPs, which are widely distributed among seed plants, are expressed in N-starved root parts and transported to the shoots, where they bind to the CEP receptor (CEPR) (Tabata *et al.*, 2014). This signal triggers the expression of class III glutaredoxins as mobile signals transported from shoots to roots via phloem to induce the expression of the NO_3_^−^ transporter NRT2.1 in the N-replete portion of the roots (Ohkubo *et al.*, 2017).

Small signaling peptides have been identified to play important roles in the regulation of root morphology, while their functions in regulating shoot architecture have received less attention. In Arabidopsis, perturbed CEP expression leads to changes in plant height and leaf shape (Roberts *et al.*, 2013). The *CEP* genes show different functions in regulating the shoot and root response to the growth conditions tested (Delay *et al.*, 2013). For example, the overexpression (ox) line of CEP2 (CEP2ox) shows fewer rosette leaves, delay of flowering, and alteration of leaf morphology in comparison to the wild type (WT); CEP3ox and CEP4ox display a similar phenotype characterized by epinasty, leaf yellowing, and reduced rosette size; CEP6ox and CEP9ox show milder changes. These results indicate that CEPs may interact with different receptors and may play distinct roles in shoot development (Delay *et al.*, 2013).

In rice, there are 17 *OsCEP* genes, and *OsCEP6.1ox* also has negative effects on rice shoot development (Sui *et al.* 2016). Compared with the WT line, *OsCEP6.1ox* transgenic lines exhibit reduced height, lower tiller number, shorter panicle length and smaller seed size (Sui *et al.*, 2016). Further functional analysis demonstrated that the regulatory activity of CEPs on panicle development may be related to the alteration of cell size but not cell number (Sui *et al.*, 2016). However, the downstream signaling components of OsCEPs and their response to N status remain largely undetermined in rice.

## Nitrogen regulation of cell division and expansion for shaping architecture

The regulation of the cell cycle by N was reported decades ago. Limited N supply suppresses DNA synthesis, cell division, cell growth, and bud growth at similar rates (Rivin and Fangman, 1980). Long-term N starvation results in the cessation of cell division and associated growth of branches in rice (Luo *et al.*, 2017). Increasing N supply levels accelerate cell division and expansion, resulting in greater biomass accumulation. Notably, the effect of the form of N supply on cell division and expansion is not significant in the short term. In Arabidopsis, the provision of either NO_3_^–^ or NH_4_^+^ causes the same effects on shoot branching (de Jong *et al.*, 2014). In rice, shoot branching is influenced significantly by the concentration but not the form of N during the vegetative stage (Luo *et al.*, 2017).

The NO_3_^−^ supply level influences the synthesis and distribution of CKs and their downstream transcription factors, which further regulate cell division in plants (Landrein *et al.*, 2018). Macadam *et al.* (1989) found that high N fertilization increased the rate of division of mesophyll cells and increased epidermal cell elongation of tall fescue leaf blades. In pea, the expression levels of cell-cycle-related genes (*PCNA*, *cyclinB*, *cdc2*, and *histone H4*) are enhanced in axillary tiller buds when the buds grow (Devitt and Stafstrom, 1995; Shimizu and Mori, 1998). N deficiency resulted in the dormancy of tiller buds, probably via altering the expression of cell-cycle-related genes (Luo *et al.*, 2017). However, it is not known whether the suppression of cell division in response to N deficiency is a direct effect of N or rather results from a signal that is transmitted to the tiller bud.

Amino acids may also influence cell division. In human tumor cells, asparagine was found to be an important regulator of amino acid homeostasis, anabolic metabolism, and proliferation (Krall *et al.*, 2016). The loss of function of asparagine synthetase (ASNS) resulted in the suppression of cell proliferation and inhibition of tumor growth in human gastric cancer cells, melanoma cells, and epidermoid carcinoma cells (Li *et al.*, 2016; Yu *et al.*, 2016). Silencing of ASNS arrested cell cycle progression at the G0/G1 phase, probably through regulation of the expression of cell cycle molecules such as CDK2 and cyclin E1 (Miao *et al.*, 2013). These results all shed light on the possible relationship between amino acids and cell division in plants. Conducting such studies may uncover new mechanisms involved in the control of shoot architecture by N.

## Nitrogen regulation of phytohormone synthesis and distribution for shaping architecture

Plants integrate internal systemic signals, such as hormones, that provide information on the N status of organs to finely adjust the growth and development of shoots and roots (Wang *et al.*, 2018a). Among these phytohormones, auxin, CKs, strigolactones (SLs), and GAs are of vital importance for regulating plant architecture. The dynamic balance between cell division and cell differentiation controls organ shape and size.

### Auxin

Fluctuation of the N supply has a significant effect on auxin distribution. A decrease in N supply commonly increases indole-3-acetic acid (IAA) accumulation in the root of plants including Arabidopsis, soybean, durum wheat, and maize (Caba *et al.*, 2000; Walch-Liu *et al.*, 2006; Tian *et al.*, 2008). The establishment of auxin distribution within plant tissues constitutes its function in plant morphogenesis, and this mainly depends on the function of auxin efflux facilitators of the PIN-FORMED (PIN) family (Friml *et al.*, 2003; Woodward and Bartel, 2005).

Plant N status may also be related to auxin synthesis and/or distribution in aboveground parts. For example, decreased NO_3_^−^ supply to rice can down-regulate the expression of multiple *OsPIN* genes and decrease ^3^H-IAA transport from shoots to roots, resulting in increased IAA content in the youngest leaves and decreased IAA content in the shoot base and roots (Sun *et al.*, 2014). In addition, the provision of a mixture of NO_3_^−^ and NH_4_^+^, in comparison to a single form of N, increases the IAA concentration in leaves and roots and increases the expression of both *OsAUX1* and *OsPIN* genes (Song *et al.*, 2011).

The molecular mechanisms of N-induced auxin distribution in shaping shoot architecture are still obscure. We have shown that N deficiency inhibits the expression of seven *OsPIN*s (*OsPIN1b/1c/2/5a/5b/9/10a*) in the roots of rice (Sun *et al.*, 2014). Since rice tiller numbers are increased by overexpression of *OsPIN2* and *OsPIN3* (Chen *et al.*, 2012; Zhang *et al.*, 2012) and are decreased by knockdown of *OsPIN10a* (Zhang *et al.*, 2012), these PIN members may be involved in N regulation of tiller bud outgrowth. However, transgenic plants overexpressing *OsPIN1* and *OsPIN5b* show inverse aboveground phenotypes (Xu *et al.*, 2005; Lu *et al.*, 2015), probably due to the disturbance of auxin-mediated bud inhibition or other secondary messengers such as CKs (Barbier *et al.*, 2019). It should be noted that auxin synthesis and distribution influenced by N supply largely depend on the plant species and N status; therefore, more evidence is required from future studies for elucidating the complex regulatory pathways of auxin for shaping N-controlled shoot architecture.

### Cytokinins

Increasing evidence indicates that elevated CK content restricts root growth and promotes shoot growth, influencing plant height, shoot branching, flowering, and seed production (Liu *et al.*, 2017). It has been shown that both the biosynthesis and distribution of CKs are closely linked to N availability during shoot and root development. Increasing NO_3_^−^ supply to barley roots can rapidly stimulate the biosynthesis and acropetal transportation of zeatin riboside (ZR), a naturally occurring CK, while NH_4_^+^ has less effect than NO_3_^−^ on the increase of ZR (Samuelson and Larsson 1993). In rice, NO_3_^−^ supply increases the concentrations of six CK forms in xylem sap, as well as leading to their high accumulation in both roots and leaves (Song *et al.*, 2013). Notably, pretreatment with either nitrate reductase or glutamine synthetase inhibitor can prevent the NO_3_^−^-simulated increase of ZR level in barley (Samuelson and Larsson 1993), while a decrease of total N concentration in tiller buds reduces the active CK content (Liu *et al.*, 2011; Ohashi *et al.*, 2017). These results suggest that entire N status or N assimilation, rather than NO_3_^−^ or NH_4_^+^ alone, determine the synthesis and distribution of CKs.

Plants possess multiple regulatory pathways of N-dependent CK biosynthesis to modulate growth. The CK synthesis gene *IPT3*, encoding adenosine phosphate-isopentenyltransferase, may play a critical role in mediating NO_3_^−^-induced CK synthesis in Arabidopsis and rice plants (Takei *et al.*, 2004; Song *et al.*, 2013) and possibly in N-controlled plant architecture. IPT3 is regulated by inorganic N sources in a NO_3_^−^-specific manner. Miyawaki *et al.* (2006) have shown that the phenotype of dramatically reduced shoot apical meristems and short, thin aerial shoots of *atipt3/5/6/7* mutants can be complemented by expressing *IPT3*. Remarkably, *IPT3* was mainly regulated by NO_3_^−^, and *ipt3* mutants failed to sense NO_3_^−^ signals to produce CKs (Takei *et al.*, 2004). NO_3_^−^-induced expression of *IPT3* is partly dependent on NRT1.1/CHL1 (Liu *et al.*, 1999; Ho *et al.*, 2009; Wang *et al.*, 2009; Kiba *et al.*, 2011). Recent findings indicate that a transcriptional regulatory system, NLP/NIGT1, controls *IPT3* and *CYP735A* gene expression in Arabidopsis in response to NO_3_^−^ (Maeda *et al.*, 2018). Nevertheless, the function of IPT3 in regulating N-controlled plant architecture at different developmental stages still needs to be investigated. In addition to IPT3, other IPT members may also be involved in N-regulated CK biosynthesis. In rice, glutamine or a related metabolite rather than NO_3_^−^ or NH_4_^+^ can enhance the expression of *OsIPT4*, *OsIPT5*, *OsIPT7*, and *OsIPT8*, with accompanying accumulation of CKs. Repressing the expression of *OsIPT4*, the dominant IPT in rice roots, significantly reduces the N-dependent increase of CKs in the xylem sap and retards shoot growth despite a sufficient N supply (Kamada-Nobusada *et al.*, 2013).

### Strigolactones

SLs have been identified more recently as a group of plant hormones that modulate plant architecture (Umehara *et al.*, 2008; Sun *et al.*, 2014; Barbier *et al.*, 2019). The function of SLs in altering shoot architecture, including involvement in plant stature, axillary tiller bud outgrowth, and tiller angle, has been partially characterized (Seto and Yamaguchi, 2014; Sang *et al.*, 2014). Small sections of stem tissue are able to supply sufficient SLs to inhibit branching in mutant shoots that are unable to synthesize SLs (Dun *et al.*, 2009), suggesting that SLs may act at very low concentrations.

Enhancement of the biosynthesis and exudation of SLs by N deficiency has been observed in several plant species (Yoneyama et al., 2007, 2012; Xie *et al.*, 2010; Sun *et al.*, 2014). In sorghum plants, limited N or phosphorus largely increases the amount of 5-deoxystrigol in the root exudates (Yoneyama *et al.*, 2007). In rice, N deficiency results in high endogenous SLs and degradation of D53 protein, a key repressor in the SL signaling pathway—the same effect as that caused by exogenous supply of the SL analogue GR24 (Sun *et al.*, 2016). These results clearly demonstrate that SLs are involved in N-regulated rice development. However, the effect of N deficiency on the synthesis of SLs depends on the plant type and experimental conditions (Yoneyama et al., 2007, 2012; Sun *et al.*, 2014). For example, SL contents in the roots of red clover and alfalfa are not significantly affected by altering the N supply (Yoneyama *et al.*, 2012).

The effect of N supply on branching in Arabidopsis is comparable between WT and mutants of SL biosynthesis (*max1* and *max3*) and signaling (*max4*) (de Jong *et al.*, 2014). Even though N limitation reduces branching in both SL mutants and WT, the mutants still produce more secondary shoots than WT under the same N-limiting condition. These results suggest that the ability to maintain N-regulated branching in Arabidopsis is at least partially dependent on SLs. In rice, the SL signaling gene *D53* can repress ideal plant architecture 1 (IPA1), a key regulator of architecture, thereby functioning as a downstream transcription factor (Song *et al.*, 2017). Thus, it is an intriguing question whether there are other targets that bind to D53 in rice plants and, if so, whether the target genes, including *IPA1*, are involved in SL participation in N-regulated plant development.

### Gibberellins

GA is involved in the regulation of the inverse relationship between plant height and tiller number. Exogenous application of GA reduces tiller number in cereal plants (Zhuang *et al.*, 2019). GA promotes plant height by stimulating the degradation of the DELLA protein SLR1 (SLENDER RICE 1) (Murase *et al.*, 2008; Sasaki *et al.*, 2003; Liao *et al.*, 2019). Since the tiller number regulator MONOCULM 1 (MOC1) relies on binding to SLR1 to avoid degradation, GAs trigger both the degradation of SLR1, leading to stem elongation, and the degradation of MOC1, leading to a lower tiller number (Liao *et al.*, 2019).

In current commonly cultivated reduced height (Rht) wheat, DELLAs are resistant to GA-stimulated destruction (Peng *et al.*, 1999), whereas the semi-dwarfism rice sd1 allele reduces the abundance of bioactive GA (Itoh *et al.*, 2002; Asano *et al.*, 2011). Notably, growth-regulating factor 4 (GRF4) can bind to GRF-interacting factor 1 (GIF1) and activate the genes related to N uptake and assimilation, while DELLA protein inhibits the binding of GRF4 to GIF1; DELLA protein accumulation thus inhibits growth and N uptake and assimilation in rice and wheat (Li *et al.*, 2018b). Moreover, N stimulation of tillering in rice is regulated by N-mediated tiller growth response 5 (NGR5) (Wu *et al.*, 2020), an APETALA2 (AP2)-domain transcription factor previously known as SMOS1 (SMALL ORGAN SIZE1) and RLA1 (REDUCED LEAF ANGLE1) (Aya *et al.*, 2014; Hirano *et al.*, 2017; Qiao *et al.*, 2017). NGR5 is a target of the GA receptor GID1; thus, NGR5 abundance is negatively associated with GA level. Mutation of NGR5 results in the insensitivity of tillering number to N supply. NGR5 regulates N-promoted H3K27me3 modification by recruiting PRC2 (POLYCOMB REPRESSIVE COMPLEX 2) to methylate the sites of *D14* (encoding Dwarf14, an SL receptor protein) and *OsSPL14* (encoding SQUAMOSA PROMOTER BINDING PROTEIN LIKE-14) and other tillering inhibition genes. Thus, in response to N supply, NGR5 inhibits the expression of the shoot-branching-inhibitory genes *D14* and *OsSPL14* and promotes tillering in rice (Wu *et al.*, 2020).

## Perspectives

**Fig. 2.**
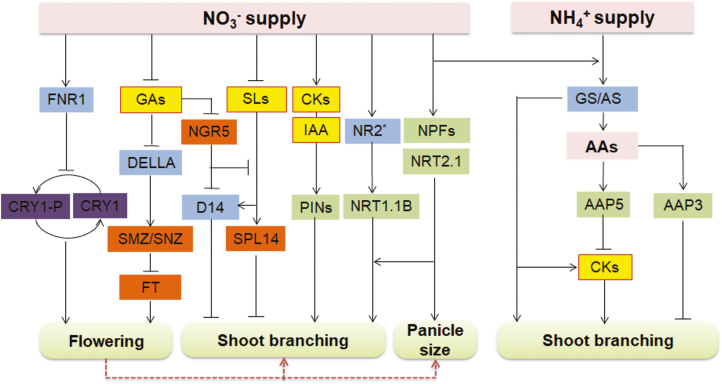
Outline of nitrogen (N) regulatory pathways altering plant architecture under conditions of nitrate (NO_3_^–^) and ammonium (NH_4_^+^) supply. NO_3_^–^ influences shoot branching, flowering time, and panicle size. NO_3_^–^ suppresses the expression of ferredoxin–NADP oxidoreductase 1 (FNR1), which modulates cryptochrome 1 (CRY1) phosphorylation and delays flowering (Yuan *et al.*, 2016). The transcription factors SCHLAFMUTZE (SMZ) and SCHNARCHZAPFEN (SNZ) can be activated by NO_3_^–^ via the gibberellic acid (GA) pathway to suppress flowering (Gras *et al.*, 2018). The flowering time can directly or indirectly alter branching and panicle structure. In addition, N supply rapidly stimulates cytokine (CK) biosynthesis and acropetal transportation (Liu *et al.*, 2011; Ohashi *et al.*, 2017). Branching is regulated by the varied distribution of auxin, which is regulated by the expression of members of the PIN-FORMED family of auxin efflux transporters (PINs) via CKs and NO_3_^–^ (Sun *et al.*, 2014). The biosynthesis of strigolactones (SLs) is suppressed by a sufficient N supply, resulting in the outgrowth of branching (Yoneyama et al., 2007, 2012; Xie *et al.*, 2010). Moreover, the expression of *indica*-type nitrate reductase 2 (NR2; indicated with an asterisk) and the nitrate transporter 1.1B (NRT1.1B) can be induced by NO_3_^–^ and enhances the absorption of NO_3_^–^ and the regulation of branching (Hu *et al.*, 2015; Gao *et al.*, 2019). Expression of the nitrate transporter 2.1 (NRT2.1) gene and putative nitrate–peptide transporter family genes (NPFs) can enhance panicle size and branching (Chen *et al.*, 2016; Huang et al., 2018, 2019b). Amino acids (AAs) influence shoot branching. The putative amino acid permease AAP3 can suppress branching, while AAP5 can alter branching by regulating the CK level (Lu *et al.*, 2018; Wang *et al.*, 2019b). Glutamine synthetase (GS), such as OsGS1.2, and asparagine synthetase (AS) can mediate the synthesis of CKs for the regulation of branching (Funayama *et al.*, 2013; Ohashi et al., 2015, 2017; Luo *et al.*, 2019). CRY1-P, phosphorylated CRY1; D14, Dwarf14 (an SL receptor); FT, flowering locus T; NGR5, nitrogen-mediated tiller growth response 5; SPL14, squamosa promoter binding protein-like 14. Arrows represent enhancement of downstream target activity. Lines with a horizontal bar at the end represent suppression of downstream target activity. Dashed lines indicate that the evidence for the regulation is not strong. Phytohormones are highlighted in yellow, transporters in green, enzymes in blue, and transcription factors in orange.

N fertilization in the field has primary effects on plant growth and development. Based on the most recent findings, we have drawn an outline of N regulatory pathways in altering flowering time, shoot branching, and panicle size under varied NO_3_^−^ and/or NH_4_^+^ supply (Fig. 2). The genes directly or indirectly involved in the N regulation of plant architecture are summarized in Table 1. It should be noted that the N regulation of different components of plant architecture and yield is affected by environmental conditions and agricultural practices. The interaction effects of planting density and N fertilization on architecture and yield are worth further investigation from both physiological and molecular genetic perspectives. In addition, the relationship between N-regulated growth duration, plant architecture, and NUE should be further investigated in crop production studies.

**Table 1.** Genes directly or indirectly involved in the nitrogen regulation of plant architecture

Gene name	Gene locus	Host plant	Protein type	Spatial expression	Transcriptional regulation by N	Effect on plant development	Reference
TaNAC2-5A	AY625683	Wheat	Transcription factor that can directly bind to the promoter regions of *TaNRT2.1-B1*, *TaNPF7.1-D1*, and *TaGS2-2A*	Mainly expressed in old leaves and flag leaves	Induced by nitrate	Promotes root and shoot growth and grain yield	He *et al.*, 2015
OsMADS57	LOC_Os02g49840	Rice	MADS-box transcription factor, interacts with OsTB1 (TEO-SINTE BRANCHED1) and targets D14 (Dwarf14)	Root stellar, sheath, leaves, shoot apical meristem	Induced by nitrate regardless of nitrate concentrations, but not by ammonium	Controls the outgrowth of axillary buds	Huang *et al.*, 2019a; Guo *et al.*, 2013
OsNRT2.3b	LOC_Os01g50820	Rice	pH-sensitive high-affinity nitrate transporter	Mainly expressed in the phloem	Induced by nitrate	High expression of *OsNRT2.3b* improves rice growth and NUE	Fan *et al.*, 2016
OsNRT1.1B	LOC_Os10g40600	Rice	Nitrate transporter	Root hair, epidermis, and vascular tissues	Induced by nitrate	Increases tiller number, grain yield, biomass, and NUE	Hu *et al.*, 2015
OsNPF7.1	LOC_Os07g41250	Rice	Member of the NPF family	Root and axillary buds	Regulated by the concentration of external N sources	Promotes axillary bud growth and increases tiller number	Huang *et al.*, 2019b
OsNPF7.4	LOC_Os04g50940	Rice	Member of the NPF family	Root and axillary buds	Regulated by the concentration of external N sources	Inhibits seedling biomass, tillering and yield	Huang *et al.*, 2019b
OsNPF7.2	LOC_Os02g47090	Rice	Low-affinity nitrate transporter	Mainly expressed in elongation and maturation zones of roots	Induced by high nitrate	Improves seedling growth, root development, and grain yield	Wang *et al.*, 2018b
OsNPF7.3	LOC_Os04g50950	Rice	Peptide transporter	Root tip, lateral root, outgrowth bud, leaf blade, stem, and panicle	Induced by organic N	Increases the number of panicles per plant, filled grain numbers per panicle, and grain N content, and enhances grain yield	Fang *et al.*, 2017
OsNPF7.7	LOC_Os10g42870	Rice	Putative nitrate transporter	Highly expressed in panicles, also in root, leaf, bud, basal part, and culm	Suppressed by high external N concentration	Promotes the outgrowth of axillary bud; increases nitrate and ammonium influx and concentration	Huang *et al.*, 2018
OsNRT2.1	LOC_Os02g02170	Rice	High-affinity nitrate transporter	Root, leaf sheath, leaf blade, internode, seed, palea, and lemma	Induced by nitrate	Increases biomass, grain yield, seed setting rate, grain number per panicle, and NUE	Chen *et al.*, 2016
SMZ	At3g54990	Arabidopsis	AP2-type transcription factor	Unknown	Induced by nitrate	Represses flowering	Gras *et al.*, 2018
SNZ	At2g39250	Arabidopsis	AP2-type transcription factor	Unknown	Induced by nitrate	Represses flowering	Gras *et al.*, 2018
FNR1	At5g66190	Arabidopsis	Oxidizes the final reduced product of the photosynthetic electron transport chain, ferredoxin, to reduce NADP^+^, resulting in ATP production	Expressed in leaf	Induced by low nitrogen conditions	Regulates expression of the circadian clock genes	Yuan *et al.*, 2016
NLP7	At4g24020	Arabidopsis	Transcription factor, NIN‐LIKE PROTEIN	Unknown	Responds to nitrate	A main regulator of nitrate signaling	Olas *et al.*, 2019
NLP6	At1g64530	Arabidopsis	Transcription factor, NIN‐LIKE PROTEIN	Unknown	Responds to nitrate	A main regulator of nitrate signaling	Olas *et al.*, 2019
OsAAP3	LOC_Os06g36180	Rice	Amino acid permease	Root, leaf, leaf sheath, culm, and panicle	Unknown	Suppresses tiller outgrowth and decreases yield	Lu *et al.*, 2018
OsAAP5	LOC_Os01g65660	Rice	Amino acid permease	Root, tiller basal part, leaf sheath, leaf blade, and young panicle	Unknown	Suppresses tiller outgrowth and decreases yield	Wang *et al.*, 2019b
OsGS1;2	LOC_Os03g12290	Rice	Glutamine synthetase	Root, basal part of shoot, leaf sheath, and leaf blade	Induced by ammonium	Reduces axillary bud outgrowth, tiller number, height, panicle number; disorder of metabolic balance and decreases grain filling	Funayama *et al.*, 2013; Ohashi *et al.*, 2015; Ohashi *et al.*, 2017
OsASN1	LOC_Os03g18130	Rice	Asparagine synthetase	Root, leaf, leaf sheath, and basal part of shoot	Induced by ammonium	Promotes tiller bud elongation and tiller number	Luo *et al.*, 2019
OsCEP6.1	LOC_Os08g37070	Rice	Mature post-translationally modified peptide of 15 amino acids	Root, shoot, lemma, palea, stamen, pistil, leaf, and panicles	Induced by low nitrogen condition	Reduces plant height, tiller number, grain number, and grain size	Sui *et al.*, 2016
OsPIN2	LOC_Os06g44970	Rice	Member of the auxin efflux carrier protein family	Root and the base of shoot	Induced by nitrate	Increases tiller number	Chen *et al.*, 2012; Sun *et al.*, 2014
OsPIN 3t (OsPIN10a)	LOC_Os01g45550	Rice	Member of the auxin efflux carrier protein	Vascular tissue	Induced by nitrate	Promotes root length and adventitious root growth; decreases effective tillers, seed setting rates, and thousand-kernel weight yield per plant	Zhang *et al.*, 2012; Sun *et al.*, 2014
OsPIN1	LOC_Os02g50960	Rice	Member of the auxin efflux carrier protein	Expressed in the vascular tissues and root primordia	Induced by nitrate	Plays an important role in auxin-dependent adventitious root emergence and tillering	Xu *et al.*, 2005; Sun *et al.*, 2014
OsPIN5b	LOC_Os08g41720	Rice	Endoplasmic reticulum-localized protein that participates in auxin homeostasis, transport, and distribution *in vivo*	Mainly expressed in panicle, culm, and leaf	Induced by nitrate	Changes auxin homeostasis, transport and distribution	Lu *et al.*, 2015; Sun *et al.*, 2014
IPT3	At3g63110	Arabidopsis	Adenosine phosphates-isopentenyl transferase	All organs in the seedlings	Responds to nitrate availability under N-limited conditions	Enhances leaf size with an increased number of cells; impairs root development	Takei *et al.*, 2004; Song *et al.*, 2013
OsNGR5	LOC_Os05g32270	Rice	Transcriptional regulator; recruits PRC2 to alter H3K27me3 methylation of targeted nitrogen-related genes	Nucleus	Increased transcription and abundance by N	Increases tiller number and grain yield	Wu *et al.*, 2020
OsGRF4	LOC_Os02g47280	Rice	Transcriptional regulator, regulates expression of multiple nitrogen-metabolism genes	Nucleus	Promoted by low N supplementation	Increases culm diameter, wall thickness, spike length, grain numbers per spike, and biomass accumulation	Li *et al*., 2018

The most important traits that influence the yield are the number of branches, panicle number, and seed size, while other aspects of plant architecture such as height, tiller angle, and leaf angle are also important for plant growth. Therefore, the trade-off among different architecture components regulated by different forms and concentrations of N should be considered for both high yield and NUE. Since the concept of “ideotype” was first put forward, the influence of environmental factors, including N fertilization, on the ideal plant architecture has received much less attention than expected, and has not been characterized in detail. To sustain the highest yield potential of the cultivars with ideotype, the architecture is expected not to be largely altered by varied N supplies in the field. Therefore, revealing the N-dependent mechanisms modulating plant architecture is helpful for molecular breeding of the ideotype with high NUE.

The influence of N fertilization on plant architecture can be monitored in real time at different scales in the field by the use of recently developed unmanned aerial vehicle (UAV)-based active canopy sensors. The modern UAV technique for providing phenotypic data shows great applicability and flexibility in the estimation of crop N status and in the analysis of plant architecture (Zaman-Allah *et al.*, 2015; Watanabe *et al.*, 2017; Elsayed *et al.*, 2018; Li *et al.*, 2018a; Buchaillot *et al.*, 2019; Cen *et al.*, 2019; Lu *et al.*, 2019). As improvement of these real-time monitoring and data-modeling techniques continues, the remote-sensing technology may be extensively applied in the future to predict the responses of the plant, including plant architecture, to N application.

To better understand the direct N regulatory pathways affecting plant architecture, identification of key quantitative trait loci and the genes controlling N-sensitive or -insensitive responses of certain components of plant architecture is expected in the future. Principal component analysis in genome-wide association studies is an effective means of extracting key information from phenotypically complex traits, and has been performed for analyzing rice architecture (Yano *et al.*, 2019). This method has been broadly used for the analysis of N-related phenotyping in some other crops (Zhang *et al.*, 2015; Monostori *et al.*, 2017; Nigro *et al.*, 2019; Steketee *et al.*, 2019) and it can be applied to isolate the key genes involved in the N regulation of plant architecture.
